# A mimic of ankylosing spondylitis, ochronosis: case report and review of the literature

**DOI:** 10.1007/s11882-021-01002-1

**Published:** 2021-03-05

**Authors:** Philip Chu, Maria C. Cuellar, Sonali J. Bracken, Teresa K. Tarrant

**Affiliations:** 1 Division of Rheumatology and Immunology, Department of Medicine, Duke University, Durham, NC 27710; 2 Division of Rheumatology, Department of Medicine, Hospital del Salvador, Santiago, Chile; 3 Department of Internal Medicine, Duke University, Durham, NC, 27710

**Keywords:** Ochronosis, Alkaptonuria, Ochronotic arthropathy, Endogenous ochronosis, Ankylosing spondylitis, Osteoarthritis, Seronegative spondyloarthropathy

## Abstract

**Purpose of Review:**

Ochronosis and alkaptonuria are manifestations of the same condition – a rare autosomal recessive disorder resulting from a constitutional lack of homogentisate 1,2-dioxygenase (HGD) with the consequent accumulation of homogentisic acid (HGA). In ochronosis, HGA undergoes autoxidation as well as enzymatic oxidation to form an ochronotic pigment that accumulates in cartilage and connective tissues. In the beginning, there is homogentisic aciduria and pigmentation of cartilages and other connective tissues. In later years, generalized osteoarthritis of the spine and large joints, termed ochronotic arthropathy, develops.

**Recent Findings:**

The diagnosis is confirmed by quantitative measurement of HGA in urine and mutation analysis of the HGD gene. One of the differential diagnoses for the skin findings is exogenous ochronosis, a limited hyperpigmentation of skin caused by some chemicals. As for the lumbar spine findings, there can be radiographic similarities with ankylosing spondylitis (AS) including reduced intervertebral disc spaces and loss of lumbar lordosis; however, ochronosis will spare the sacroiliac joint, and the lumbar spine will show dense, wafer-like disk calcification with a vacuum disc phenomenon and broad syndesmophytes.

**Summary:**

Here, we present a case of a patient with probable ochronosis that was treated many years as ankylosing spondylitis without response, and we provide a review of the current literature on ochronosis pathogenesis, diagnosis, and treatment.

## Introduction

Ochronosis and alkaptonuria are manifestations of the same condition resulting from an inborn error of tyrosine metabolism. Alkaptonuria is an autosomal recessive mutation resulting in a deficiency of homogentisate 1,2-dioxygenase (HGD) that leads to an accumulation of HGA. The accumulation of HGA and its byproducts in connective tissues, leads to the ochre-like pigmentation in the skin, sclera, and cartilage – hence the term ochronosis. The abundant urinary excretion of HGA, darkens slowly upon oxidation to air. This oxidation is hastened by the addition of and avidity to alkali. Thus, the urine findings are termed alkaptonuria [1].

The history of ochronosis goes back centuries. Alkaptonuria has been noted as far back as 1584 by Scribonius who first observed urine “as black as ink.” In 1866, Virchow described ochronosis – noting yellow-brown pigmentation on intervertebral discs, articular cartilage, and arteriosclerotic plaques. In 1902, Albrecht recognized the connection between ochronotic arthropathy and alkaptonuria. In 1908, Garrod recognized this disease as one of the “inborn errors of metabolism” [1, 2].

Ochronosis is a rare autosomal recessive disorder with an estimated prevalence of 1:250,000 to 1:1,000,000 [3, 4], but higher frequencies have been reported in the Dominican Republic and Slovakia [5–7]. The gene is composed of 14 exons and maps to chromosome 3q13.33. At least 203 HGD gene variants associated with alkaptonuria have been identified. The most frequent European variant is the M368V mutation in exon 13. The missense variant G161R was most frequent in patients from Slovakia, and the missense variant A112V was most common in patients from Jordan [8]. Patients carry homozygous or compound heterozygous variants of the *HGD* gene. All variants identified in alkaptonuria patients are summarized in the *HGD* mutation database (http://hgddatabase.cvtisr.sk/).

## Case report

**Fig. 1:**
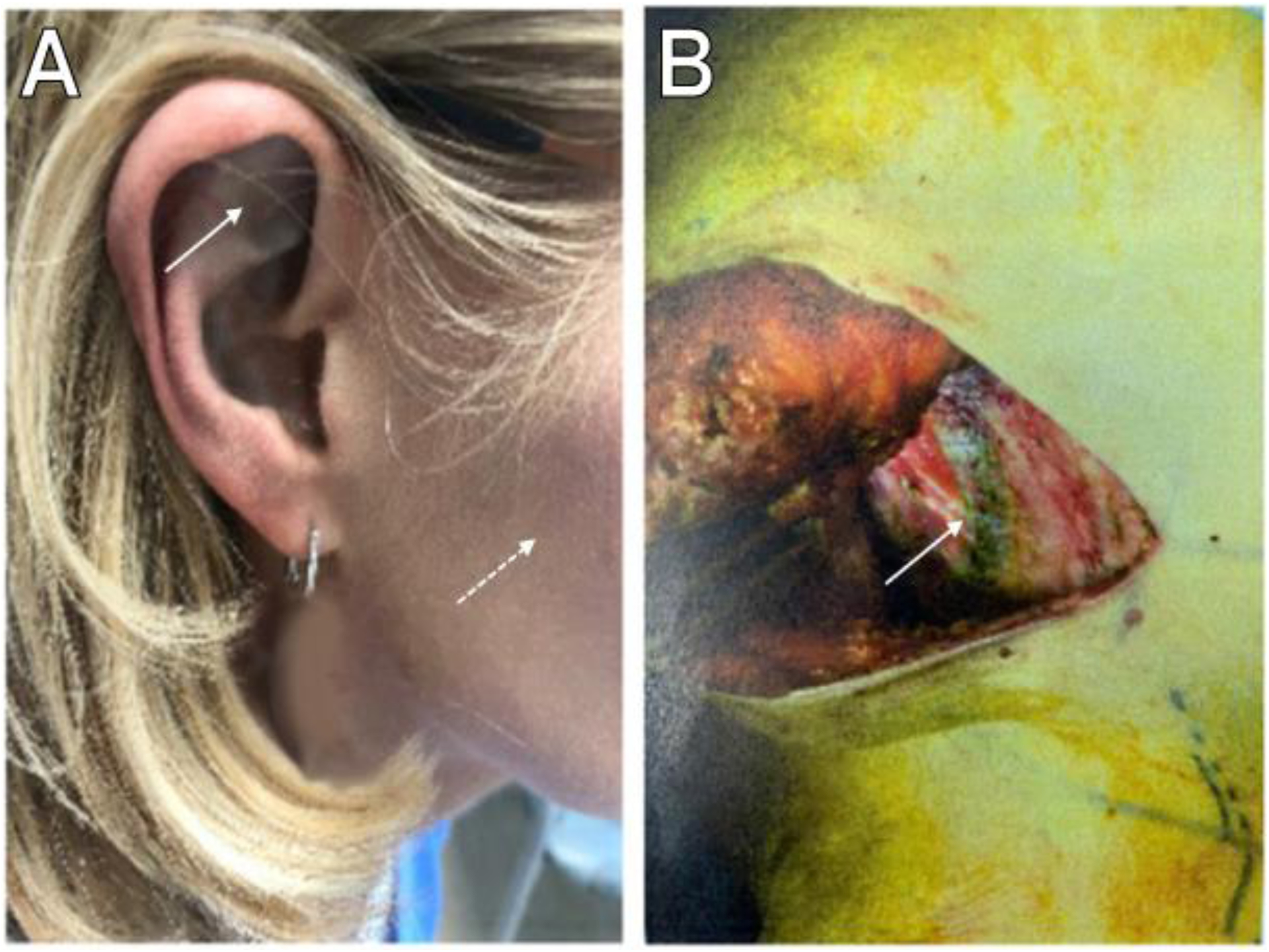
Physical findings in Caucasian female with suspected ochronosis. Bluish discoloration around pinnae and outer ears, sparing the ear lobes (1A; solid arrow) and facial skin (dashed arrow). Bluish colored ribs in a thoracic surgery (1B; solid arrow).

The patient is a 48 years old Caucasian female with a history of chronic back pain for 10 years who was previously diagnosed with ankylosing spondylitis; however, her symptoms had been refractory to treatment with NSAIDS and TNF-alpha antagonists. She described progressive worsening of spinal stiffness and bilateral knee pain. At the time of the consultation, she was being treated with secukinumab and prednisone with no clinical improvement. Review of symptoms was also positive for mild dyspnea. Physical exam revealed bluish discoloration around the palpebral fissures, pinnae, and outer ears, sparing the ear lobes (Fig. 1A), blue-tinged dental enamel and nail folds/plates, a systolic crescendo-decrescendo murmur, and a mild restriction of lumbar flexion. There was no synovitis. Two years prior, patient had removed breast silicone implants, at which time her plastic surgeon commented that “your ribs are blue, and I took pictures of them” (Fig. 1B).

Laboratory data revealed a normal blood count, liver tests, and biochemical profile. Erythrocyte sedimentation rate (ESR) was normal, 20 mm/h; C reactive protein (CRP) was normal, 1.2 mg/L. Antinuclear antibody level was present, but anti-Ro, -La, -double stranded DNA, and -Smith were negative. C3, C4, and a urinalysis were normal. Echocardiogram and chest computed tomography (CT) scan did not show any significant pathology. Although the clinical suspicion of ochronosis was high, neither the genetic test, the serum HGA level, nor the lumbar image were done since the patient refused the tests.

## Pathogenesis

**Fig. 2:**
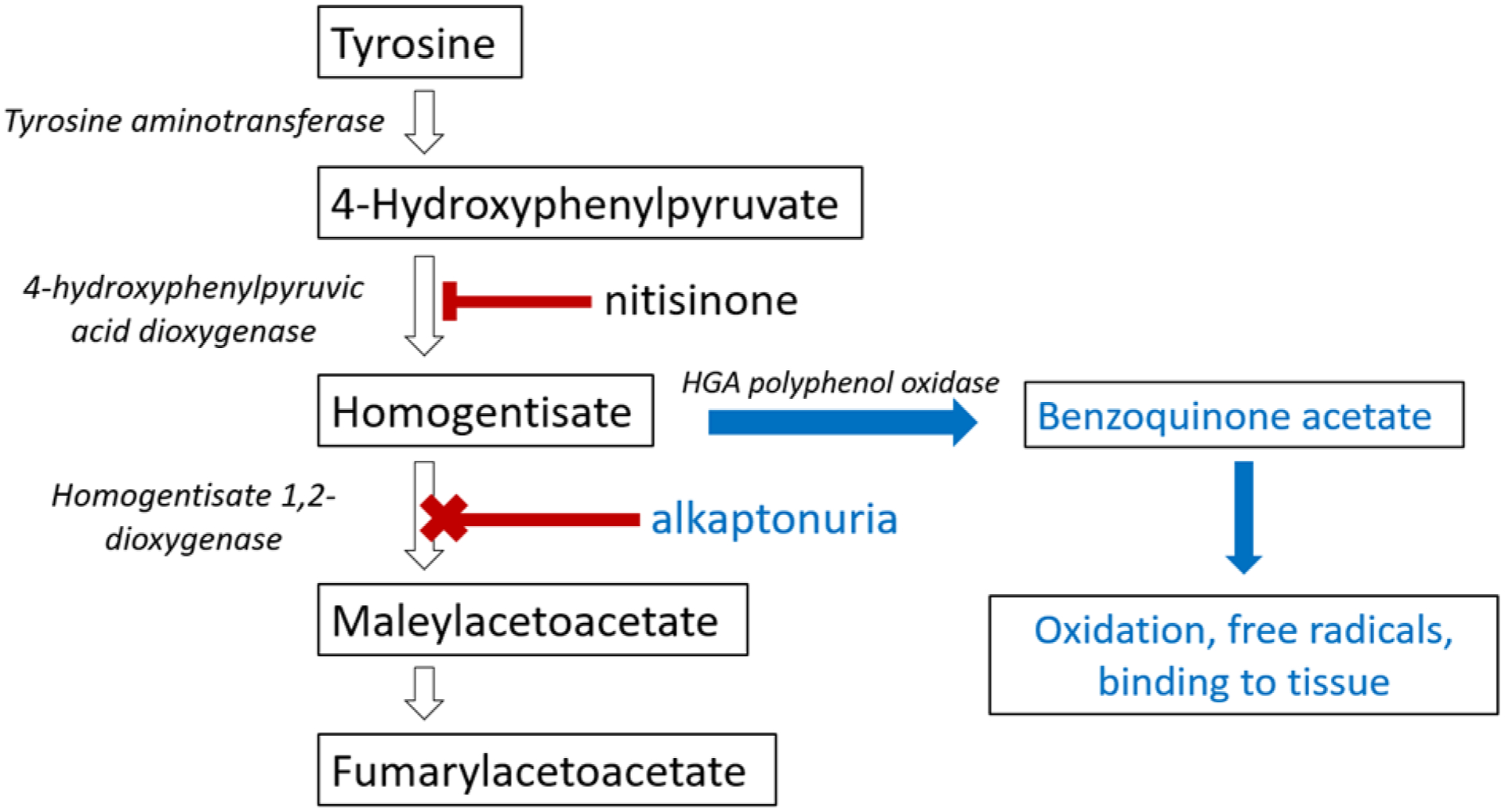
Pathway of tyrosine and homogentisic acid metabolism leading to pathology in alkaptonuria (ochronosis). The tissue accumulation of homogentisic acid and benzoquinone acetate is believed to be responsible for the clinical manifestations of ochronosis. Adapted from reference [4].

The clinical manifestations and arthropathy of ochronosis can be attributed to the accumulation of HGA and its oxidized metabolite, benzoquinone acetic acid. In the tyrosine degradation pathway, tyrosine is normally converted in the liver through several enzymatic steps into maleylacetoacetic acid. However, in ochronosis, a deficiency of HGD leads to an accumulation of HGA. Accumulation of HGA can be converted by homogentisic acid polyphenol oxidase into benzoquinone acetic acid, which can lead to oxidation, free radical formation, and tissue binding (Fig. 2) [4]. High levels of HGA and its oxidized byproducts can affect collagen crosslinking, leading to increased cartilage stiffness and structural weakness. Cell growth can also be inhibited in a concentration-dependent manner [1].

## Clinical presentation

The typical triad of ochronosis consists of dark urine on addition of alkali, ochronotic pigmentation of the connective tissues, and arthritis. Alkaptonuria can be present at birth and is often noted by the discoloration of urine staining the diapers; however, this clinical feature may be absent or missed, and the patient may go undiagnosed for numerous years. In a case series of patients, 21% were diagnosed before 1 year of age, but the mean age of diagnosis was 29 years [4]. Differential diagnoses to be considered in the evaluation of discolored urine are included in Table 1.

Chronic joint pain may develop between the third and fourth decades of life. Arthropathy affects the spine and large joints, with frequent low back pain. Symptoms in the lumbar and thoracic spine occur before the cervical spine. The spine involvement may mimic ankylosing spondylitis with a reduced Schober’s test, but arthropathy spares the sacroiliac joints, and sacroiliitis is not present on imaging (Table 2).

Peripheral arthritis occurs later, and involves large- and medium-sized joints, with relative sparing of the small joints of the hands and feet. Common features include pain, stiffness, crepitus, and contractures. The average age of joint replacement was 53 years in patients with ochronosis compared to the national average of 67 years for patients with OA [9]. The knee is the most commonly affected peripheral joint, estimated in 64% of ochronosis patients, and detected after age 40, many years after onset of symptoms in the spine [10]. The hip and pelvis are involved in 35% of cases but detected typically after age 50. The shoulder is involved in 43% of cases. Tendons can be symmetrically involved, and mimic enthesopathy, and increase the risk of rupture from even minimal trauma [10]. In one study, radiographic severity scores were present at age 30 and increased linearly with age [4].

In the third decade of life, skin discoloration can be seen as bluish-black pigmentation of the pinnae of the ears, sclera, cornea, and conjunctiva. Pigment deposition can also lead to hardening of the pinnae [10].

**Fig. 3:**
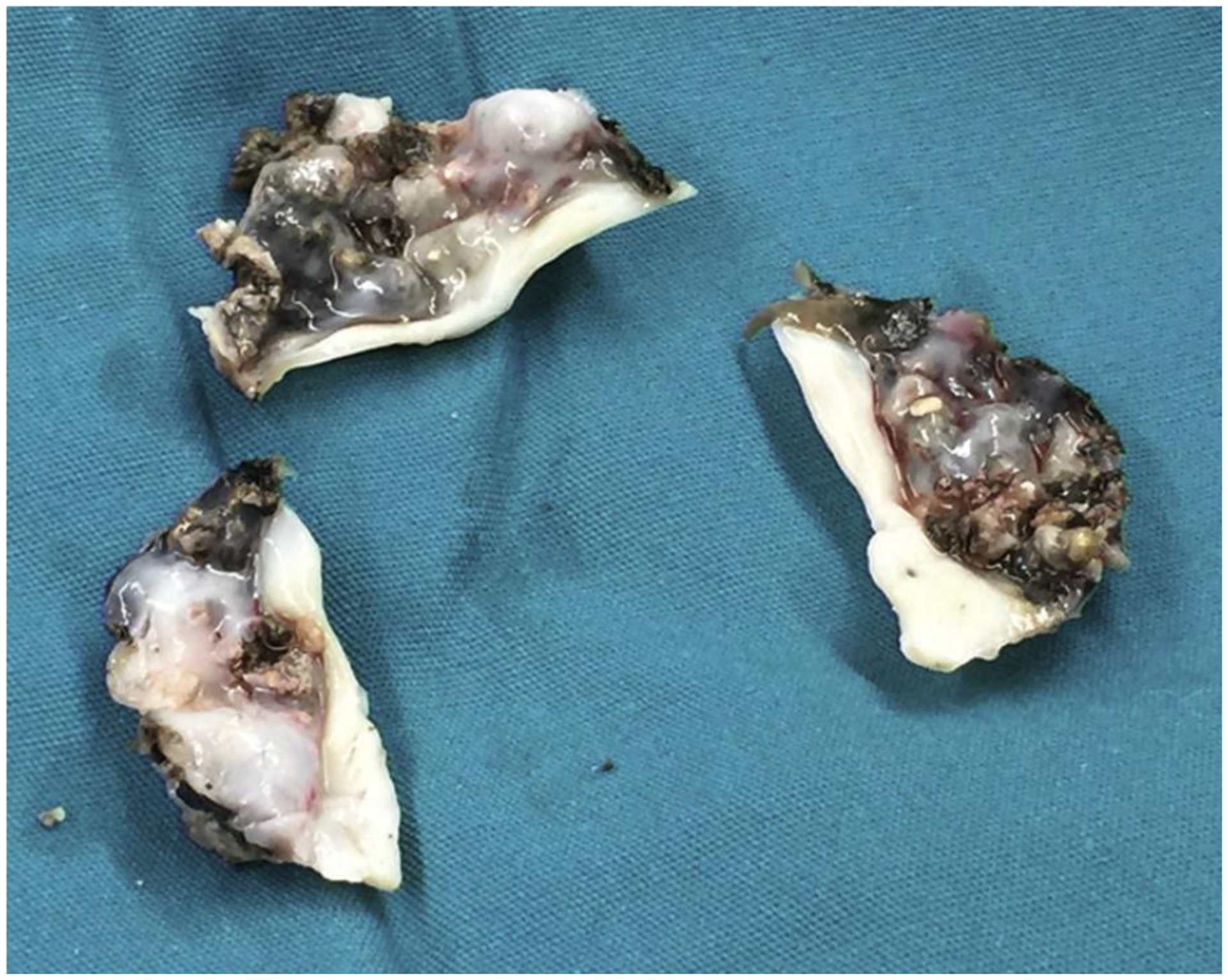
Aortic valve in patient with ochronosis. Reproduced with permission [11].

The cardiovascular system is also involved although estimates are unknown. Ochronotic pigment in the valve may lead to dystrophic calcification and stenosis over time. Figure 3 shows aortic valve leaflets calcified, thickened, and pigmented in a patient with aortic valve stenosis due to ochronosis [11]. Aortic or mitral valve calcification was detected at a mean age of 54 [4]. Unfortunately, biological valve prosthesis with recurrent ochronotic deposition has been reported, although the recurrence rate of ochronosis on bioprosthetic valves is not known [12]. While most patients did not have coronary-artery calcification before the age of 40, half of patients have CT evidence of coronary-artery calcification by age 59 [4].

Ochronosis can be suspected when a patient presents with early onset arthritis and when there is bluish black pigmentation of the sclerae or the cartilage of the eyes, ears, and/or nose. Prior to making the diagnosis of endogenous ochronosis, one should first consider the possibility of exogenous ochronosis from topical creams as well as discoloration from systemic medication side effects (Table 1).

## Laboratory evaluation

Darkening of the urine in alkaptonuria can be seen with prolonged exposure to air as well as addition of alkali (i.e. sodium hydroxide or ferric chloride). The diagnosis is confirmed by measurement of 24-hour urinary excretion of HGA, measured by gas chromatography [13]. Testing of urinary HGA by capillary gas chromatography can be obtained in the urine organic acids screen via mayocliniclabs.com (Test ID: OAU).

DNA testing for genetic mutations of the *HGD* gene for homogentisate 1,2-dioxygenase can be performed [4] when medication effects and other differential diagnoses for discolored urine and skin are negative. Genetic testing for the *HGD* gene can be obtained via Invitae.com (Test code: 06140).

In a case series of patients with ochronosis, the mean plasma homogentisate level was 6.6 micrograms/mL, with a range of 3.0–27.8 micrograms/mL (normal: undetectable). The mean 24 hour urinary homogentisate levels were 3.12 mmol/mmol creatinine, with a range of 0.4 and 12.4 mmol/mmol creatinine (normal <0.017 mmol/mmol creatinine). The mean plasma tyrosine level was 1.4 mg/dL (normal: 1.2 mg/dL). Only 1 of 58 patients had reduced creatinine clearance. One patient had elevated alanine aminotransferase level. The mean hemoglobin level was 13.9 g/dL. Eleven out of 58 patients had elevated ESR [4, 13]. It is important to emphasize that 25% of ochronosis patients may have elevations in the ESR as this could skew practitioners toward an incorrect diagnosis of seronegative spondyloarthropathy.

## Imaging

**Fig. 5:**
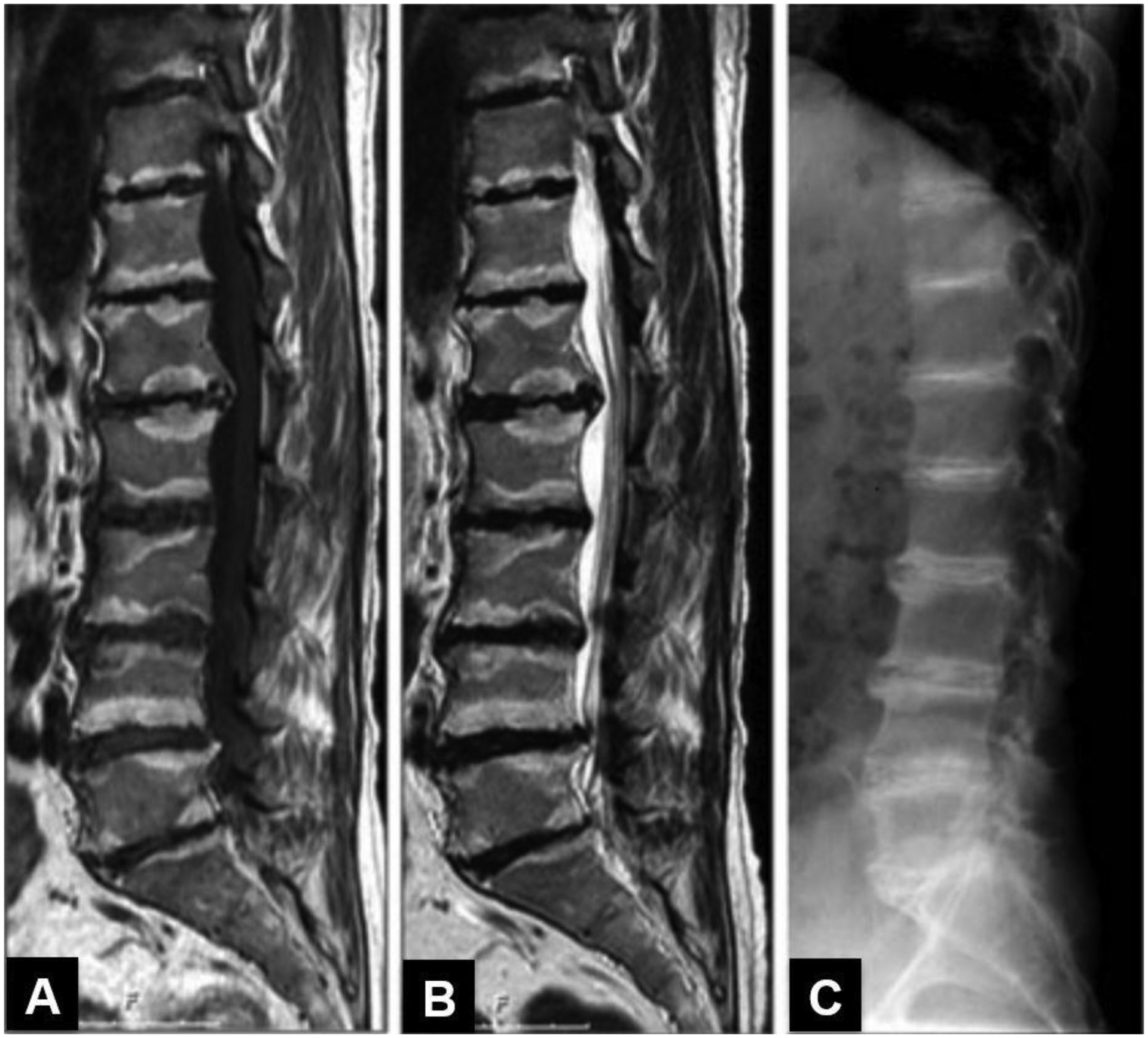
50- year- old man with ochronosis. Magnetic resonance imaging (MRI) (a and b) and radiography (c) of the lumbar spine, with classic changes of ochronosis. Reproduced with permission [15].

In the spine, disc calcification is the most specific finding of ochronosis. Some publications describe the lumbar spine as having “wafer-like disc calcification.” Other principal findings are narrowing of the intervertebral spaces and ostial bridges (Fig. 5) [10, 14]. Both plain radiographs and CT of the spine may show annular hyperdense regions due to disc calcification. There are also intervertebral osteophytes and vacuum phenomenon – the latter refers to air in the disc seen as hypodensity in the anterior part of the intervertebral disc.

MRI of the spine shows low signal on both T1 and T2 weighted images due to loss of hydration of the nucleus pulposus, calcification, and global loss of intervertebral disc height. Atypical diffuse homogeneous modic type 2 (fatty) end plate changes are visible, too. The diffuse type 2 modic end plate changes are unusual, and ochronosis should be considered in the differential diagnosis when these changes are present (Fig. 5) [15, 16]. Herniated disc, ligamentum flavum hypertrophy, thickening of anterior and posterior longitudinal ligaments are degenerative changes visualized on MRI [16].

**Fig. 6:**
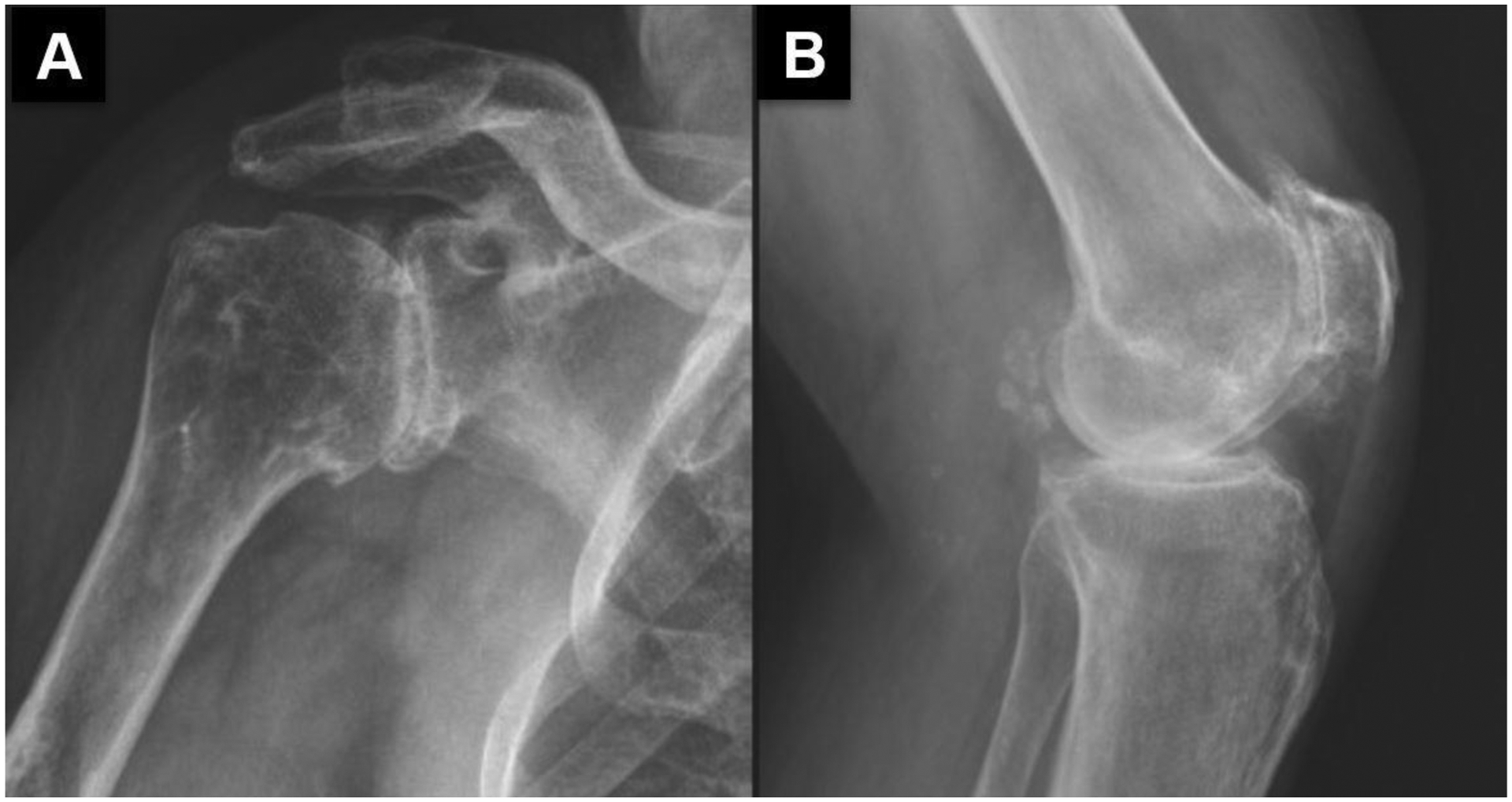
Radiographs of the ochronotic patientś right shoulder (a) and left knee (b) showing severe osteoarthritis, chondrocalcinosis is present in the popliteal bursa. Reproduced with permission [33].

In the knee, synovial effusion may be present, mimicking an inflammatory arthropathy, which combined with elevated ESR and/or lumbar findings may mislead practitioners toward an incorrect diagnosis of seronegative spondyloarthropathy. Radiographs reveal retropatellar joint space narrowing due to degeneration of the femoral cartilage [10]. Otherwise, characteristic findings of osteoarthritis, including joint space narrowing, subchondral sclerosis, new bone formation are also seen in ochronosis (Fig. 6) [17].

The hip and pelvis are affected in 35% of cases. The ochronotic pigment causes calcification and fragmentation of the cartilage, which leads to narrowing of the joint space [10, 14]. Complete degeneration may even lead to disappearance of the femoral head [18]. Sacroiliac joints are rarely involved [1], which may be one of the most important distinguishing features from seronegative spondyloarthropathies, such as ankylosing spondylitis and psoriatic arthritis.

The shoulder is affected in 43% of cases (Fig. 6). The degeneration of the humeral head leads to decreased joint space, and superior migration of the humeral head [10]. As ochronosis pigmentation can deposit on tendons, radiographic tendon calcification can also be seen [1], which might be confused with enthesitis if not carefully evaluated.

## Histopathology

Histologic examination of the bone and soft tissue reveals ochronotic pigmentation, thickened synovium, and reactive giant cells [10, 19].

In the joint space, synovial effusion due to inflammation of the cartilage may be present in about 50% of patients [20]. The inflamed synovial fluid contains hyperplastic synoviocytes, fibrosis, and pigment-containing macrophages [1, 10]. While the synovium may be similarly hyperplastic and inflamed in rheumatoid arthritis or seronegative inflammatory arthritis, pigment containing macrophages would not be expected. Pigmentation of synovium can be seen in pigmented villonodular synovitis and in amiodarone with long term use [21], but the inflammatory synovitis and macrophage infiltration would be less likely.

## Treatment

Nitisinone (Orfadin) is being investigated as a treatment for ochronosis [22–25]. Nitisinone inhibits the enzyme 4-hydroxyphenylpyruvic acid dioxygenase, the second enzyme in the tyrosine catabolic pathway, and was approved by the FDA in 2002 for the treatment of hereditary tyrosinemia. In alkaptonuria, the drug reduces serum and urine HGA concentrations. In mouse models, lifetime treatment resulted in reduction of plasma HGA and prevented ochronosis [22], whereas midlife administration arrested further deposition but did not reverse existing pigment deposition [23]. In humans, a 3-year randomized control trial demonstrated that nitisinone (2 mg/day) reduced plasma and urine HGA by 95% and had relatively few adverse effects [24]. None of the 18 nitisinone-treated patients lacking signs of aortic stenosis at baseline progressed to aortic sclerosis. In contrast, 7 of 17 control patients lacking aortic stenosis at baseline developed aortic stenosis, suggesting that nitisinone impeded progression [24]. A phase III efficacy study “Suitability of Nitisinone in Alkaptonuria 2” (SONIA2) randomized 138 patients to assess the reduction of HGA and progression of alkaptonuria (clinicaltrials.gov, ID: NCT01916382). The study is estimated to be completed in February 2020 with analyses pending. Another clinical trial “Subclinical Ochronotic Features in Alkaptonuria” (SOFIA) will evaluate patients of different ages to determine the optimal age at which to begin treatment with nitisinone [25]. Nitisinone may have potential teratogenic effect and would need to be held before and during pregnancies [26].

Vitamin C (up to 1g/d) can suppress urinary excretion of benzoquinone-acetic acid at various doses ranging from 500 mg twice a day to 10 g/day [26, 27]; however, the long-term efficacy has not been proven. *In vitro* tests have suggested the benefit of anti-oxidant effects of ascorbic acid and N-acetylcysteine [28].

Restriction of dietary protein, namely tyrosine and phenylalanine, is controversial. Although it can lower HGA excretion, long-term effects have not been studied. Dietary compliance is also very difficult and may risk nutritional deficiencies [26].

## Outcomes

While ochronosis is not known to reduce life expectancy, major comorbidities regarding arthritis, cardiovascular sequelae, and skin pigmentation severely impact the quality of life of patients starting in the 3rd decade of life [29]. Recognizing the disease and distinguishing it from other inflammatory and non-inflammatory forms of arthritis are important to identify patients for treatment options directed toward this specific metabolic pathway. With an understanding of the metabolic pathways, availability of genetic testing, and new treatments being investigated, major debilitating comorbidities can hopefully be prevented down the road.

## Conclusion

- Ochronosis/alkaptonuria is an autosomal recessive mutation resulting in a deficiency of homogentisate 1,2-dioxygenase, leading to an accumulation of HGA.
- While alkaptonuria can be present at birth, other symptoms of skin discoloration, progressive arthritis, valvular heart disease occur after age 20 due to ochronotic pigment deposition.
- Ochronosis frequently involves the spine and can mimic ankylosing spondylitis radiographically and clinically but spares the sacroiliac joint.
- Nitisinone reduces serum and urine HGA concentrations and may be a therapy to treat patients with hereditary ochronosis/alkaptonuria; thus, correctly distinguishing this metabolic disease from seronegative spondyloarthropathy, osteoarthritis, and exogenous ochronosis is important for practitioners to recognize.

## Figures and Tables

**Fig. 4:**
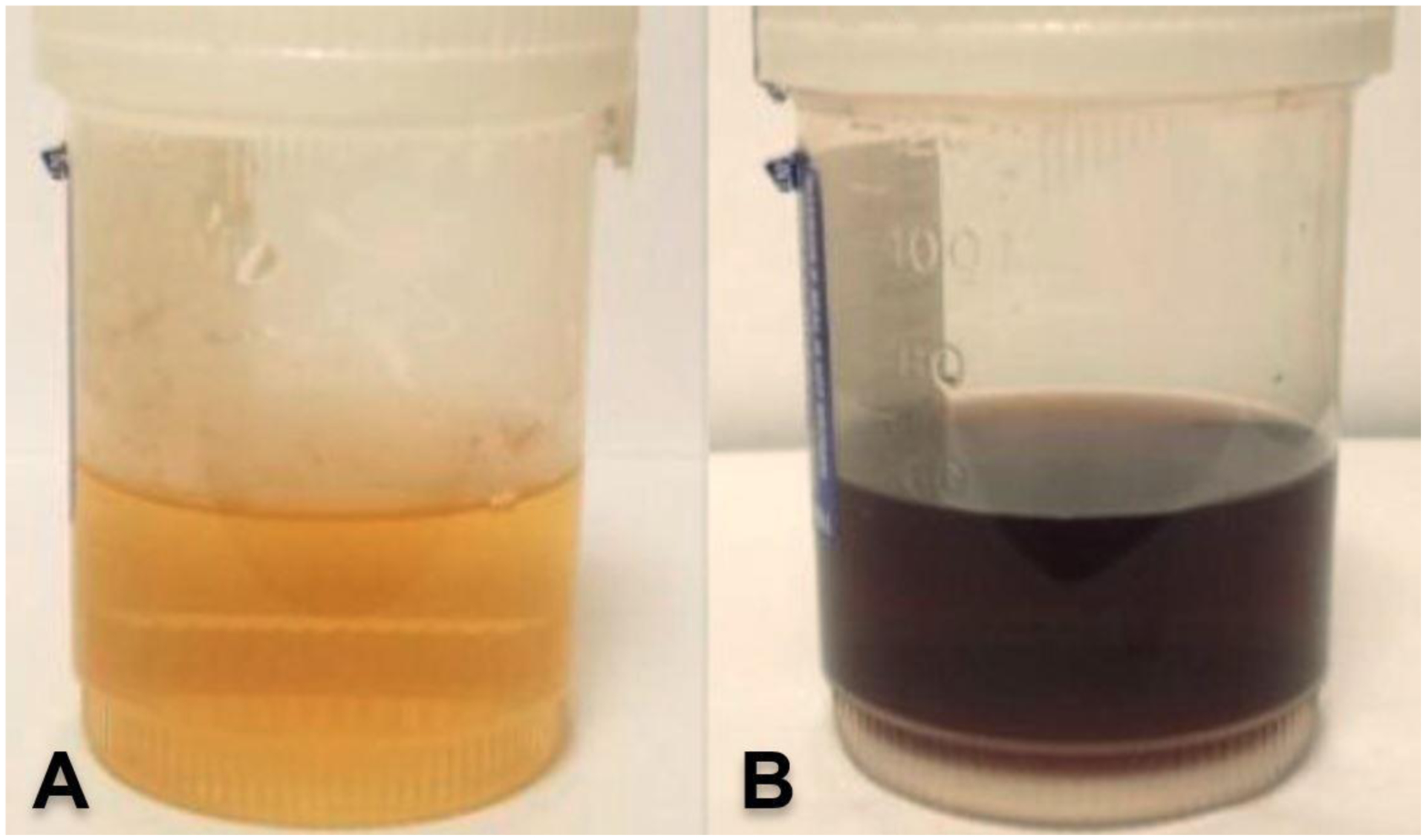
Photographs of the urine sample in a patient with ochronosis before (a) and after (b) alkalization. Reproduced with permission [33].

**Table 1:** Differential diagnosis of ochronosis and alkaptonuria

Clinical Feature	Differential diagnosis
Spine and peripheral joint arthropathy [3]	Ankylosing spondylitis
	Diffuse idiopathic skeletal hyperostosis
	Hemochromatosis
	Hyperparathyroidism
	Calcium pyrophosphate deposition disease
	Amyloidosis
	Pigmented villonodular synovitis
	Amiodarone [21]
Dark urine discoloration [30]	Porphyria
	Myoglobinuria
	Hemoglobinuria
Discoloration of skin and cartilaginous structures of the face [31, 32]	Topical phenols
	Topical hydroquinone skin-lightening cream
	Oral minocycline
	Amiodarone
	Levodopa
	Hydroxychloroquine

**Table 2:** Comparison of ochronosis and ankylosing spondylitis

	Ochronosis	Ankylosing spondylitis
Joints involved	Spine = Knees = Hips	Spine > Hips, Knees
Sacroiliac joints	Not involved	Involved
Disk calcification	Dense, wafer-like	Mild to moderate
Vacuum disks	Common	Rare
Syndesmophytes	Broad	Vertical
